# Variable Interhemispheric Asymmetry in Layer V of the Supplementary Motor Area following Cervical Hemisection in Adult Macaque Monkeys

**DOI:** 10.1523/ENEURO.0280-20.2020

**Published:** 2020-10-09

**Authors:** A. Contestabile, R. Colangiulo, M. Lucchini, E.M. Rouiller, E. Schmidlin

**Affiliations:** 1 Department of Basic Neuroscience, University of Geneva, Genève CH-1205, Switzerland; 2 Department of Neurosciences and Movement Sciences, Section of Medicine, Faculty of Sciences and Medicine, Fribourg Center of Cognition, University of Fribourg, Fribourg CH-1700, Switzerland

**Keywords:** corticospinal projections, interhemispheric asymmetry, non-human primate, pyramidal neurons, spinal cord injury, supplementary motor area

## Abstract

Motor cortical areas from both hemispheres play a role during functional recovery after a unilateral spinal cord injury (SCI). However, little is known about the morphologic and phenotypical differences that a SCI could trigger in corticospinal (CS) neurons of the ipsilesional and contralesional hemisphere. Using an SMI-32 antibody which specifically labeled pyramidal neurons in cortical Layers V, we investigated the impact of a unilateral cervical cord lesion on the rostral part (F6) and caudal part (F3) of the supplementary motor area (SMA) in both hemispheres of eight adult macaque monkeys compared with four intact control monkeys. We observed in F3 (but not in F6) interindividual variable and adaptive interhemispheric asymmetries of SMI-32-positive Layer V neuronal density and dendritic arborization, which are strongly correlated with the extent of the SCI as well as the duration of functional recovery, but not with the extent (percentage) of functional recovery.

## Visual Abstract

**Figure:**
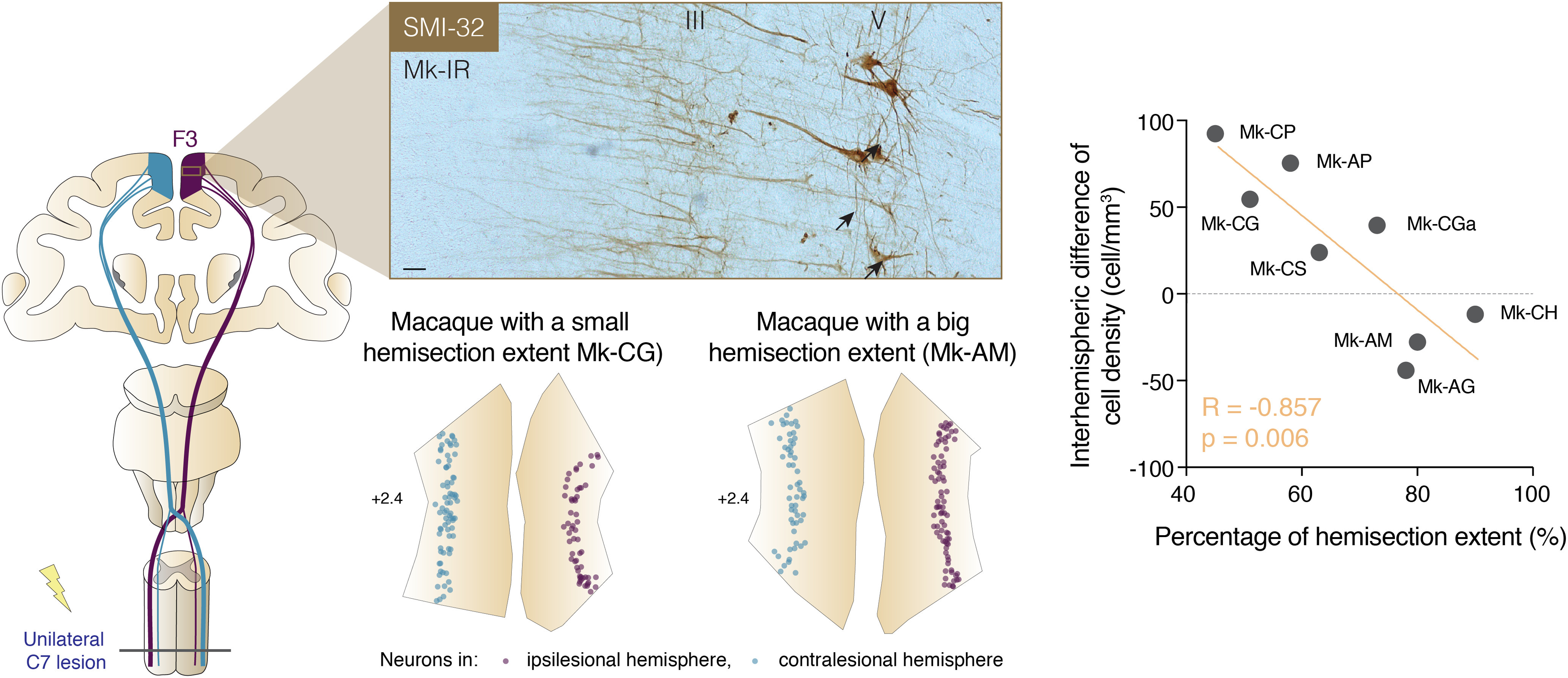

## Significance Statement

This study consists in a precise quantification on two different levels of the histological consequences on the long term of a traumatic and sudden unilateral interruption of the corticospinal (CS) tract at cervical level in eight non-human primates (adult macaque monkeys). The lesion affected the density and the morphology of Layer V pyramidal neurons in the supplementary motor area (SMA), in the form of an interhemispheric adaptive asymmetry, correlated to the lesion size and duration of functional recovery. These changes are reminiscent of those observed in SMA after unilateral lesion of the primary motor cortex (M1), suggesting to some extent comparable mechanism of functional motor recovery from unilateral cortical or spinal lesion. The dendritic arborization in the basal dendrites of the SMI-32-positive neurons in Layer V showed a more prominent interhemispheric effect of the lesion than the apical dendrites.

## Introduction

In non-human primates, the hand area of the primary motor cortex (M1 or F1) is subdivided in an old M1 and a new M1 (Rathelot and Strick, 2009). The new M1 is at the origin of the corticomotoneuronal (CM) projection, representing the anatomic support of manual dexterity, a prerogative of primates (Lawrence and Kuypers, 1968; Courtine et al., 2007; Lemon, 2008; Yoshida and Isa, 2018). Although M1 is the main contributor to the corticospinal (CS) projection (including the CM projection), non-primary motor areas such as the premotor cortex (PM), the SMA (SMA-proper or F3) and the cingulate motor areas (CMA) are also at the origin of CS projections (Luppino et al., 1994; Rouiller et al., 1994, 1996; Dum and Strick, 1996). In particular, SMA projects to the cervical spinal cord, where the motoneurons controlling hand (fingers) motor function are located (Jenny and Inukai, 1983). There is evidence that part of the CS projection from SMA may be CM (Rouiller et al., 1996), but the influence of SMA on hand motoneurons is functionally less strong than the one of M1 (Maier et al., 2002; Boudrias et al., 2010). The multiple representations of the hand in several motor cortical areas (M1, PM, SMA, CMA) of primates is the basis for a vicarious scenario of functional redistribution of hand function control in case of selective and focal lesion affecting a motor structure. For instance, after unilateral lesion of the hand area in M1 functional recovery, although often incomplete, depends on plasticity of intact non-primary motor areas, such as PM (Liu and Rouiller, 1999; Dancause et al., 2005; Hoogewoud et al., 2013; Orczykowski et al., 2018) and/or SMA (McNeal et al., 2010; Morecraft et al., 2015). Although such rearrangement of the cortical motor circuits is believed to occur mostly in the ipsilesional hemisphere, there is still controversy about the role played by the contralesional hemisphere (for the non-human primate, see Morecraft et al., 2016; Savidan et al., 2017). In a recent article from this laboratory, we reported that a unilateral lesion of the M1 hand area led to a variable interhemispheric asymmetry in the detection of Layer V pyramidal neurons in SMA, identified with the marker SMI-32 (Contestabile et al., 2018). This anatomic variable interhemispheric imbalance possibly reflects an adaptive interhemispheric contribution of the bilateral SMA to recovery, depending on the lesion size as well as on the duration of functional recovery (Contestabile et al., 2018). It was argued that these observations may represent a putative anatomic support of diaschisis, originally defined as a “loss of function and electrical activity in an area of the brain because of a lesion in a remote area that is neuronally connected with it” (Finger et al., 2004; see also von Monakow, 1914). In other words, because of a unilateral M1 lesion, the function of the SMA is affected at distance differently on the ipsilesional versus contralesional hemispheres, because the reciprocal connections between M1 and SMA are stronger ipsilaterally than contralaterally, in the intact state. More recently, the concept of diaschisis has been extended to a structural dimension, in the form of “connectional diaschisis,” focused on postlesion changes of connectivity in networks/circuits directly or indirectly related to the focal lesion site (Carrera and Tononi, 2014).

**Figure 1.**
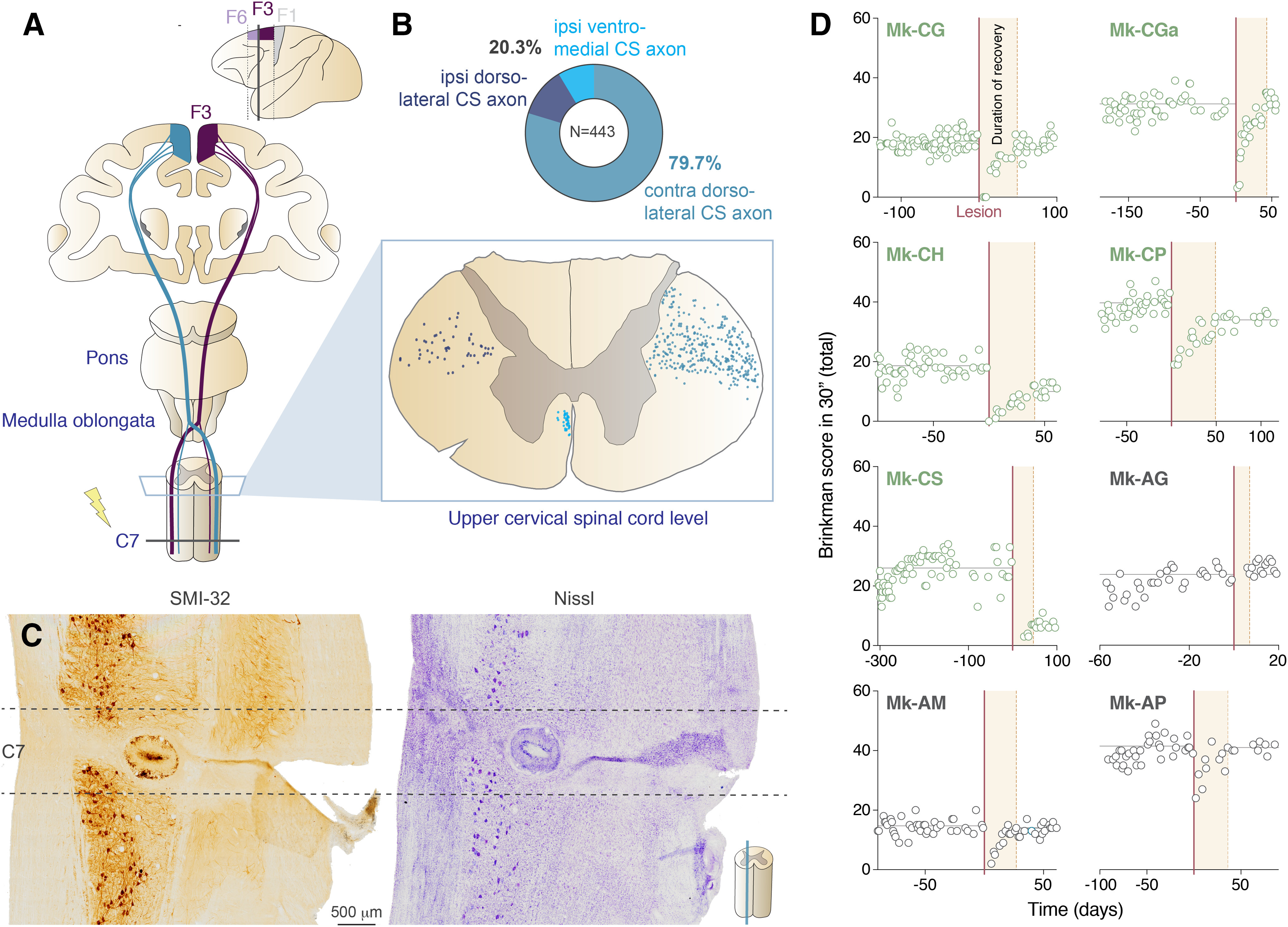
CS projection of SMA. ***A***, upper panel, Schematic representation of a macaque brain showing the location of pre-SMA (area F6), SMA-proper (area F3), and M1. Lower panel, Schematic representation of the CS projections of F6 (bilateral). ***B***, lower panel, Upper cervical spinal cord section showing the localization of CS axons labeled by a unilateral BDA injection in the SMA. Upper panel, Pie chart reporting the percentage of BDA-positive CS axons present in the ipsilateral ventromedial, ipsilateral dorsolateral, and contralateral dorsomedial part of the spinal cord. The majority of BDA positive CS axons are located in the contralateral dorsolateral part of the spinal cord. ***C***, Photomicrographs of sagittal histologic sections of the spinal cord of a lesioned animal (Mk-CS) showing the induced permanent lesion at the C7/C8 level. The histologic sections derived from two different series processed to visualize SMI-32 staining (left) or Nissl staining (right). Scale bar: 500 μm. ***D***, Graphical representation of behavioral performance in the modified Brinkman board task of the macaque monkeys included in this study (SCI in green and SCI + anti-Nogo-A treatment in dark gray). The manual performance of the ipsilesional hand is given by the score (number of pellets retrieved in the first 30 s of the task from the randomly distributed wells) as a function of time (days) before and after a SCI. Day 0 corresponds to the day of the lesion (vertical red line). Prelesional and postlesional average of the scores are indicated with horizontal gray lines. The total duration of functional recovery of manual dexterity after lesion is given by the time interval between the lesion (red vertical line) and the onset of the postlesion plateau (brown area). *Figure Contributions*: E. M. Rouiller and E. Schmidlin performed and supervised the experiments on the monkeys and analyzed the data.

In the current study, the main goal was to transpose this concept of interhemispheric adaptable SMA reorganization related to functional recovery from a lesion of M1 (Contestabile et al., 2018) to a cervical spinal cord hemisection. Does a unilateral spinal cord injury (SCI) also affect differently the contralesional versus the ipsilesional SMA, because the CS projection from SMA is predominantly crossed (Fig. 1*A*,*B*)? Following a hemisection of the cervical cord in macaque monkeys, it was observed that the CS axotomy did not lead to a retrograde death of CS neurons in M1 (Wannier et al., 2005) but, instead, to a somatic shrinkage of CS neurons in Layer V of the contralesional M1 (Beaud et al., 2008). As far as the functional recovery from CS tract injury or cervical cord lesion is concerned, the underlying mechanisms are multiple and complex, involving plasticity at spinal, subcortical and cortical levels, depending also on the time course of rehabilitative training (Galea and Darian-Smith, 1997; Freund et al., 2007; Nishimura et al., 2007; Nishimura and Isa, 2009, 2012; Zaaimi et al., 2012; Sugiyama et al., 2013; Isa and Nishimura, 2014; Chao et al., 2019). At cortical level in particular, following unilateral cervical lesion, the bilateral M1 areas are involved in the functional recovery at early stage whereas, at later stage, the contralesional M1 area plays a major role, together with the bilateral PM (Freund et al., 2007; Nishimura et al., 2007; Nishimura and Isa, 2012; Isa and Nishimura, 2014). Moreover, a recent study identified two distinct cortical network dynamics that are implicated in the recovery of a unilateral SCI: the grasping-related intrahemispheric interactions from the contralesional PM to the contralesional M1, and motor-preparation-related interhemispheric interactions from the contralesional to ipsilesional PM (Chao et al., 2019).

What happens in SMA Layer V bilaterally after cervical hemisection and functional recovery? One may hypothesize that CS neurons in SMA also survive (as those in M1: see above), but their phenotype is likely to be modified (connectional diaschisis), in a variable manner depending on the hemisphere (because of the asymmetric strength of its CS projection), as well as on lesion parameters, such as lesion extent, as well as on functional recovery parameters (duration of recovery, extent of recovery). To test these different hypotheses, SMI-32-labeled neurons in the bilateral SMAs were analyzed histologically in eight macaque monkeys subjected to cervical hemisection and compared with intact monkeys (*n* = 4), based on the assumption that graded changes in the phenotype of CS neurons in SMA impacts on the detection of those neurons using the marker SMI-32 (Contestabile et al., 2018; see also Geyer et al., 2000).

## Materials and Methods

The methods to analyze the histologic sections in SMA following SCI were similar to those reported in detail in a recently published open access article (Contestabile et al., 2018), also focused on SMA but following a lesion of the M1. Nevertheless, for the sake of convenience, the methods are reminded here, although in a somewhat shorter version.

### Macaque monkeys

The present histologic analysis in SMA was conducted on 12 adult macaque monkeys (10 *Macaca fascicularis*, two *Macaca mulatta*; Table 1). All procedures were conducted in accordance with guidelines of the federal and local (cantonal) veterinary authorities (veterinary authorizations: 157e/04, 175/04, 187/05, 188/06, 193/07). All 12 monkeys included in the current study were already reported in previous articles addressing distinct issues related to C7 spinal cord lesion (Freund et al., 2006, 2007, 2009; Beaud et al., 2008, 2012; Hoogewoud et al., 2013) or as intact control animals (Contestabile et al., 2018). Eight animals (Mk-CG, Mk-CGa, Mk-CH, Mk-CP, Mk-CS, Mk-AG, Mk-AM, and Mk-AP) were subjected to a unilateral cervical cord lesion at C7 level, for whom detailed and comprehensive experimental data were reported previously, such as lesion characteristics, behavior, plasticity of CS projection system in M1, and at spinal cord level, effects of the anti-Nogo-A antibody therapy (Freund et al., 2006, 2007, 2009; Beaud et al., 2008, 2012; Hoogewoud et al., 2013). Four other monkeys (Mk-IC, Mk-IE, Mk-IR, and Mk-IZ) had no lesion (intact monkeys) and were used as controls; they already appeared as such in a recent study (Contestabile et al., 2018). Three out of eight cervical cord lesioned monkeys were treated with anti-Nogo-A antibody (Mk-AG, Mk-AM, and Mk-AP). Comprehensive descriptions of the anti-Nogo-A antibody treatment procedure were published earlier (Freund et al., 2006, 2007, 2009; Beaud et al., 2008, 2012; Hoogewoud et al., 2013). In short, the anti-Nogo-A antibody treatment (14.8 mg) was applied during for four weeks immediately after the SCI. The anti-Nogo-A antibody was delivered from an osmotic pump, placed in the back of the animal. The other five monkeys subjected to SCI received a control antibody (Freund et al., 2006, 2007, 2009). At the end of the behavioral assessments (see below), the animals were euthanized under deep anesthesia obtained with an intraperitoneal overdose of pentobarbital sodium (90 mg/kg body weight), as previously reported (Freund et al., 2006, 2007, 2009; Beaud et al., 2008, 2012; Hoogewoud et al., 2013).

**Table 1:** Summary of the individual properties of each monkey

		Intact				Untreated					Treated with anti-Nogo-A antibody		
		Mk-IC	Mk-IE	Mk-IR	Mk-IZ	Mk-CG	MK-CGa^3^	Mk-CH	Mk-CP	Mk-CS	Mk-AG^3^	Mk-AM	Mk-AP
General information	Birthday	17.07.99	15.06.96	02.02.04	12.05.96	20.02.01	21.05.03	20.02.01	22.12.97	30.04.97	28.03.02	20.02.01	09.03.98
	Sex	M	M	F	M	M	M	M	F	M	M	M	F
	Species	Fasc	mul	Fasc	Fasc	fasc	fasc	fasc	fasc	mul	fasc	fasc	fasc
	Date of killing	17.08.09	15.02.02	22.12.09	09.02.04	13.01.05	19.01.07	07.02.05	09.11.04	21.09.01	03.08.05	14.02.05	16.11.04
Lesion	Date of lesion	-	-	-	-	25.08.04	16.08.06	29.09.04	04.05.04	07.03.01	13.04.05	29.09.04	02.06.04
	SCI side	-	-	-	-	left	right	right	left	left	left	right	left
	Weight at time of lesion (kg)	-	-	-	-	5.1	-	4.1	3.8	4.0	3.7	4.5	4.2
	Age at the time of the lesion (d)	-	-	-	-	1282	1183	1317	2325	1407	1112	1317	2277
	Hemisection extent (%)	-	-	-	-	51	73	90	45	63	78	80	58
	Degree of functional recovery from SCI, total score (%)^1^	-	-	-	-	90	100	53	83	22	100	96	99
	Duration of functional recovery (d)^2^	-	-	-	-	49	43	41	49	47	7	27	36
Treatment	type of antibody	-	-	-	-	ctrl	ctrl	ctrl	ctrl	ctrl	ATI	ATI	11c7
	Cocentration (mg/ml)	-	-	-	-	9	7	9	3.7	3.7	3.6	9	3.7
	Volume (ml)	-	-	-	-	4	2	4	4	4	2	4	4
	Days of antibody injection	-	-	-	-	30	29	28	29	32	28	28	15

M = male; F = female; fasc = *M. fascicularis*; mul = *M. mulatta*; rhe = *Rhesus*.

1 Expressed in percentages of postlesion total score at plateau divided by prelesion total score in the modified Brinkman board task: all slots.

2 Time interval from the day of lesion to the beginning of postlesion plateau, as defined by Kaeser et al. (2011).

3 Mk-CGa and Mk-AG suffered from a more caudal lesion than C7/C8. These monkeys were taken into account in this study but with some reserves.

### Behavior

As recently reported (Contestabile et al., 2018), manual dexterity was quantified in the eight monkeys subjected to unilateral SCI, based on the modified Brinkman board task, testing the precision grip (opposition of thumb and index finger), needed to grasp small food pellets in 25 vertically oriented slots and 25 horizontally oriented slots, randomly positioned over a Perspex board (Schmidlin et al., 2011). After habituation to the housing facility and transfer to a primate chair using positive reinforcement (http://www.unifr.ch/neuro/rouiller/home/nhp), the macaques monkeys were progressively trained daily to perform the modified Brinkman board task, separately for each hand, until they reached a prelesion plateau of performance. The duration of the training period and the plateau level of performance was variable across monkeys, as reported earlier based on a large cohort of animals (Kaeser et al., 2014). Then, at plateau, the monkeys were tested daily, once for each hand, during a prelesion phase of several weeks to establish a score of manual dexterity of reference in the intact monkey. The same behavioral test procedure was pursued also daily after the SCI, without additional training besides the test itself. Based on the postlesion tests, it was possible to assess the progressive functional recovery from the SCI and the performance level of the postlesion plateau.

The dramatic drop of score (usually to zero) immediately after the lesion and the deficits of precision grip following such cervical lesion have been reported in detail earlier (Freund et al., 2006, 2007, 2009; Hoogewoud et al., 2013) and are summarized in Figure 1*D*. After unilateral C7 injury, a progressive although incomplete functional recovery was observed until reaching a plateau of motor performance a few weeks after the SCI (Freund et al., 2006, 2007, 2009; Hoogewoud et al., 2013; Fig. 1*D*). The behavioral parameter of interest here was the percentage of functional recovery for the ipsilesional hand, given by the average total score (number of pellets retrieved from both vertical and horizontal wells in 30 s) at postlesion plateau divided by the average total score at prelesion plateau (Freund et al., 2006, 2007, 2009; Hoogewoud et al., 2013; Fig. 1*D*). Moreover, the plots of manual dexterity scores as a function of time were used to define the time duration until the postlesion plateau was reached (duration of functional recovery; Fig. 1*D*).

### Unilateral cervical cord lesion

The surgical procedure to perform the unilateral lesion was described in detail in previous reports (Freund et al., 2006, 2007, 2009; Beaud et al., 2008, 2012; Hoogewoud et al., 2013), an experimental procedure summarized below. Anesthesia was induced by an intramuscular injection of ketamine (Ketalar; Parke-Davis; 5 mg/kg), and atropine was injected intramuscularly (0.05 mg/kg) to reduce bronchial secretions. Before surgery, the analgesic Carprofen was delivered (Rymadil, 4 mg/kg, s.c.). Then, deep and stable anesthesia was obtained via a continuous perfusion (0.1 ml/min/kg) through an intravenous catheter placed in the femoral vein of a mixture of 1% propofol (Fresenius) and a 4% glucose solution. The monkey was positioned in a stereotaxic headholder in a ventral decubitus position, allowing the spinal processes from C2 to Th1 to be exposed, preceding a complete C6 laminectomy and an upper C7 hemilaminectomy. The dorsal root entry zone at the C7/C8 border was then identified, providing a medial landmark for placing a surgical blade (no. 11; Paragon), which was inserted vertically 4 mm in depth to generate an incomplete section of the cervical cord at C7 level. In most cases, such a section completely interrupted the CS tract in the dorsolateral funiculus unilaterally. Following the SCI, the monkey was kept alone in a separate cage for a couple of days to monitor its health condition and provide specific postoperative care (antibiotics, analgesics).

### Histology and neuroanatomical material for analysis

The general histologic and analysis methods are similar to those recently reported (Contestabile et al., 2018) and repeated in a shorter version below. After euthanasia, the spinal cord segment (C3-T4) comprising the SCI was cut parasagittally in either three or five series of, respectively, 50 or 30 μm sections and processed to visualize either BDA, Nissl or SMI- 32 staining (Fig. 1*A–C*). The extent of the cervical cord lesion was measured in consecutive longitudinal sections (scar tissue and absence of neuronal staining) and transposed in a transversal reconstruction to assess the percentage of lesion with respect to the entire corresponding hemicervical cord (white and gray matter).

**Figure 2.**
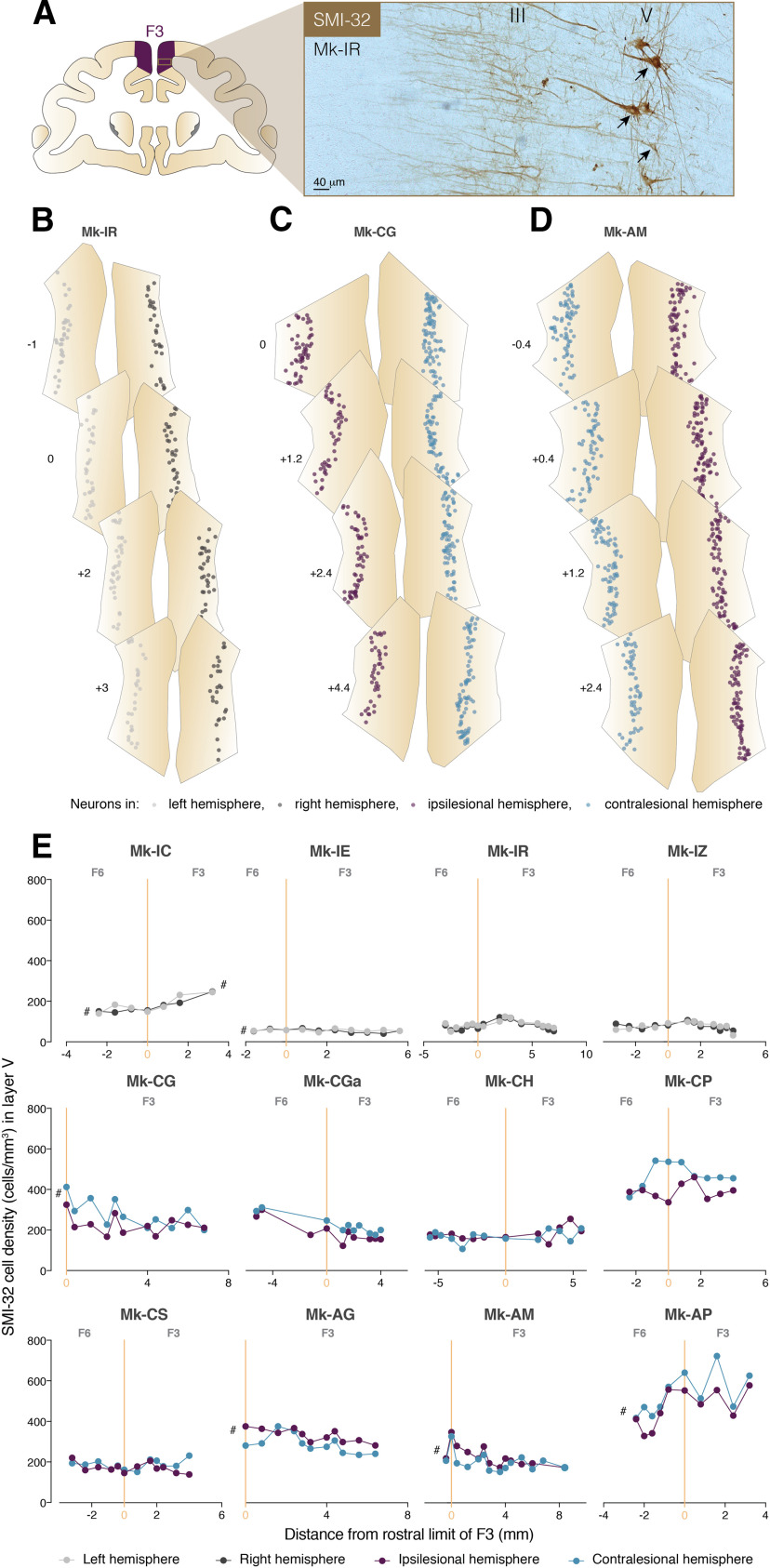
Neuronal density in the Layer V of SMA in intact and SCI monkeys. ***A***, Photomicrograph of coronal brain histologic section of an intact macaque monkey (Mk-IR) stained with SMI-32 and magnified in F3 (scale bar: 40 μm). Layers III and V are visible and SMI-32-positive pyramidal neurons are indicated with arrows. ***B–D***, Localization of SMI-32-positive Layer V neurons in bilateral F3 and F6 areas of three representative macaque monkeys (intact: Mk-IR, SCI: Mk-CG and SCI + anti-Nogo-A treatment: Mk-AM). Four sections per macaque monkeys are shown and the relative rostro-caudal position of the sections from the F3-F6 border is reported in millimeters. Negative distance values belong to F6 and positive distance values belong to F3. ***E***, Graphs representing the rostro-caudal gradient (from F6 to F3) of SMI-32-positive cell density in Layer V of all monkeys (intact: Mk-IC, Mk-IE, Mk-IR, and Mk-IZ; SCI: Mk-CG, Mk-CGa, Mk-CH, Mk-CP, and Mk-CS; SCI + anti-Nogo-A treatment: Mk-AG, Mk-AM, and Mk-AP). The cell density for each hemisphere is plotted as a function of the distance from the F3-F6 border, which has been set to 3 mm rostrally to the genu of the arcuate sulcus. Negative distance values belong to F6 and positive distance values belong to F3. The symbol # was used to indicate that the analyzed cortex region was not complete (sections lacking for the analysis). *Figure Contributions*: E. M. Rouiller and E. Schmidlin performed the experiments on the monkeys and generated the histologic sections. A. Contestabile and R. Colangiulo performed the microscopic analysis of the histologic sections. A. Contestabile analyzed the data.

In all monkeys (SCI and intact), the brain was cut in the frontal plane into 50-μm-thick frozen sections with both hemispheres facing each other on the same slide (Fig. 2*A*). Sections were collected in five or eight consecutive series, one of them immunoreacted to visualize the marker SMI-32 (Sternberger and Sternberger, 1983), as previously reported (Liu et al., 2002; Wannier et al., 2005; Beaud et al., 2008). Frontal brain sections were examined under bright-field illumination (at a total magnification of 200×). Brain regions of interest (mostly SMA) were vectorized using Neurolucida 9 (MBF Bioscience) with a computer-interfaced Olympus BX40 microscope (Olympus Schweiz AG), a computer-controlled motorized stage (Märzhäuser Wetzlar GmbH, type EK 32 75×50) and a digital camera (Olympus U-PMTVC). The Photomicrographs captured with a digital camera were processed using the CorelDraw software (color, brightness, and contrast were not modified) and then quantification was conducted using the software Neuroexplorer (MBF Bioscience; for example, see Fig. 2*B–D*). At that step, the investigator of the histologic material was blinded against information on animal group (intact vs SCI) and side of the cervical cord hemisection. The size of the SCI expressed as a percentage of hemisection extent were reported previously (Freund et al., 2006, 2007, 2009; Beaud et al., 2008, 2012; Hoogewoud et al., 2013) and are reminded in Table 1. SMA and its two subdivisions (F3 and F6, respectively, corresponding to SMA-proper and pre-SMA) in both hemispheres was delineated from M1 or PM laterally and from cingulate motor area (CMA) ventrally, based on cytoarchitectural landmarks (Liu et al., 2002). SMI-32-positive neurons were counted in SMA Layers V, and the surface of the delineated F3 or F6 was calculated in relation with the defined limits. Based on an exhaustive plotting method (Fregosi and Rouiller, 2017; Fregosi et al., 2017, 2018), the cellular density in Layer V of F3, respectively, F6, was computed on individual sections. It is the number of identified SMI-32-positive cells in that layer in one hemisphere divided by the corresponding volume of SMA (given by the thickness of the section multiplied by the area-of-interest). The criteria used to include a neuron were the same proposed in our previous article (Contestabile et al., 2018): (1) SMI-32-positive; (2) the soma, the nucleus or the nucleolus and at least four proximal dendrites of the neuron have to be identifiable; and (3) the neuron is located in the cortical Layer V. The criteria to set the rostrocaudal limit between F3 and F6, as well as the precise territories of F3 and F6 analyzed were the same as in Contestabile et al. (2018).

### Interhemispheric difference of cell density (IDCD)

**Figure 3.**
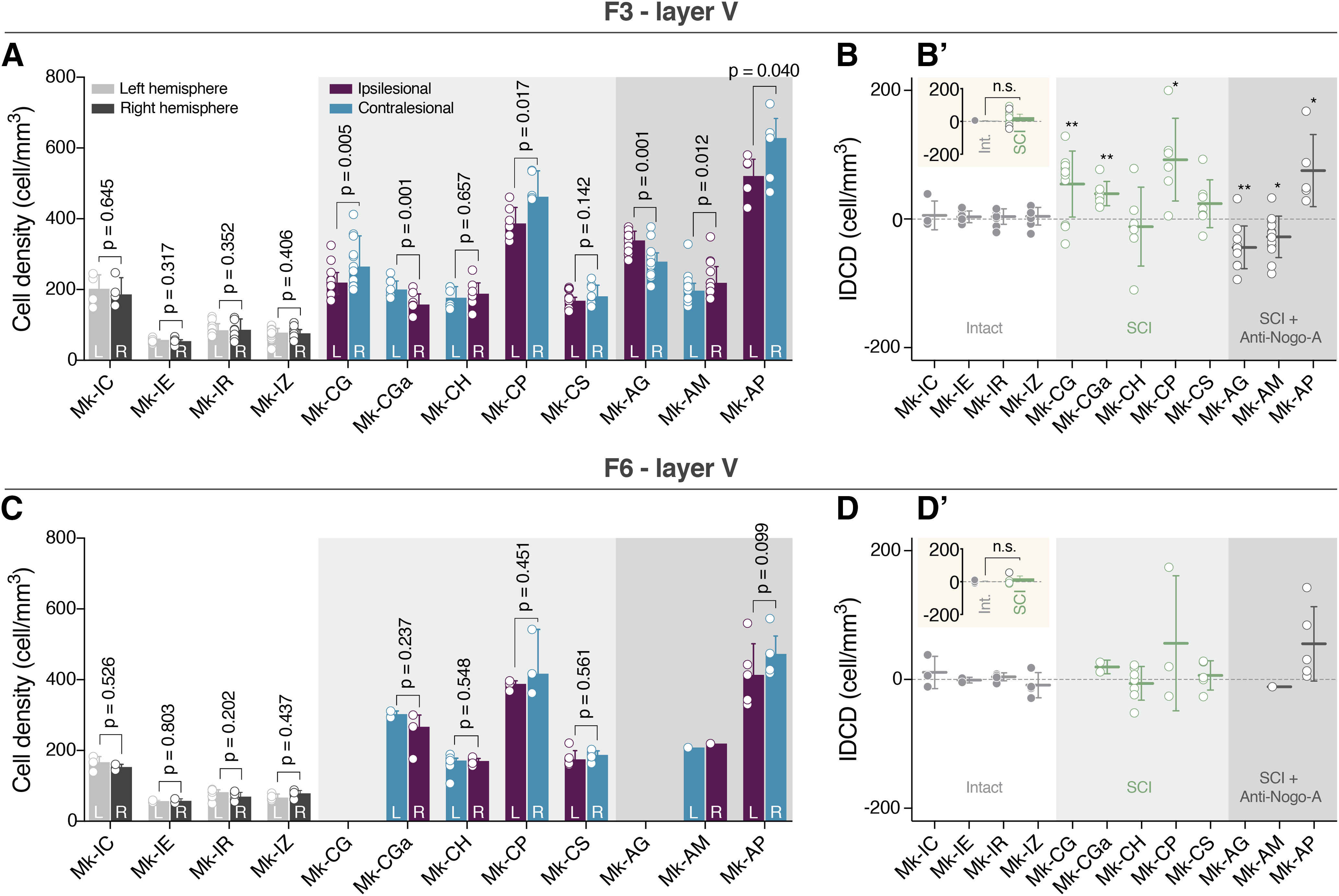
IDCD after SCI in F3 and F6. ***A***, Histograms reporting the cell density of Layer V SMI-32-positive neurons in F3 for intact (white background), SCI (light gray background), and SCI-treated monkeys (dark gray background). For each couple of histograms are reported the side (L = left and R = right) and the location in function of the SCI (Ipsi = ipsilesional and Contra = contralesional). As statistical test, a paired *t* test or Wilcoxon test was performed (*p* values are reported) comparing the cell density in the two hemispheres in each consecutive histologic section. ***B***, Histograms showing the IDCD of Layer V SMI-32-positive neurons of F3 for intact (white background), SCI (light gray background), and SCI-treated monkeys (dark gray background). A positive IDCD corresponds to an ipsilesional bias in pyramidal cell density, while a negative IDCD corresponds to a contralesional bias in pyramidal cell density. ***B’***, upper left inset, Comparison of calculated IDCD in F3 between groups. No statistical difference is observed (unpaired *t* test: *t*_(10)_ = 0.8276, *p* = 0.4272). ***C***, Histograms reporting the cell density of Layer V SMI-32-positive neurons in F6 for intact (white background), SCI (light gray background), and SCI-treated monkeys (dark gray background). For each couple of histograms, are reported the side (L = left and R = right) and the location in function of the SCI (Ipsi = ipsilesional and Contra = contralesional). As statistical test, a paired *t* test or Wilcoxon test was performed (*p* values are reported) comparing the cell density in the two hemispheres in each consecutive histologic section. ***D***, Histograms showing the IDCD of Layer V SMI-32-positive neurons of F6 for intact (white background), SCI (light gray background), and SCI-treated monkeys (dark gray background). ***D’***, upper left inset, Comparison of calculated IDCD in F6 between groups. No statistical difference is observed (unpaired *t* test: *t*_(7)_ = 1.084, *p* = 0.3142). For ***A***, ***C***, the median and the interquartile range are indicated. For ***B***, ***D***, the mean ± SD are reported. L = left hemisphere, R = right hemisphere, *p ≤ 0.05, **p ≤ 0.01). *Figure Contributions*: E. M. Rouiller and E. Schmidlin performed the experiments on the monkeys and generated the histologic sections. A. Contestabile and R. Colangiulo performed the microscopic analysis of the histologic sections. A. Contestabile analyzed the data.

An IDCD in SMA Layer V was computed in each histologic section by subtracting the SMI-32-positive neuron density in the ipsilesional SMA (relatively intact side) from the SMI-32-positive neuron density in the contralesional SMA (strongly connected with the SCI; Fig. 3*B*,*D*). Positive IDCD means that more SMI-32 neurons were found in SMA strongly connected to the side where the SCI was performed, whereas negative IDCD corresponds to a larger number of SMI-32 neurons in Layer V of SMA homolateral to the SCI. In intact monkeys, IDCD was expected to be close to zero, whereas in monkeys subjected to SCI, the IDCD was hypothesized to diverge from zero (Fig. 5*A*, e.g., gray line). As for our previous work (Contestabile et al., 2018), the interhemispheric comparison was made on each individual section, so that the mirrored SMA territories analyzed on each side had comparable area and position.

### Single SMI-32-labeled neuron analysis

To investigate whether the unilateral SCI impacted on the microstructure of single SMI-32-positive neurons in SMA Layer V, in particular on their dendritic trees, a so-called Sholl analysis was performed, identical to the one reported recently (Contestabile et al., 2018). The sampling and inclusion criteria were similar: three SMI-32-positive pyramidal neurons of Layer V per hemisphere per section were analyzed in four different sections (12 neurons per hemisphere in total) in each of five representative monkeys: two intact monkeys and three monkeys with SCI. The following criteria were applied to specifically select SMI-32-positive neurons: (1) to be representative of the entire region-of-interest, one neuron was picked in each of the dorsal, middle, and ventral parts of the medial wall in F3; and (2) at least intact primary and secondary dendrites and an apical dendrite reaching the Layer III without interruption had to be clearly identified. Two distinct regions of the dendritic tree of each SMI-32-positive neurons were analyzed: the basal dendrites excluding the axon and the apical dendrite.

### Statistics

Statistical analysis was conducted with GraphPad Prism 7. The normality of sample distributions was assessed with the Shapiro–Wilk criterion. In each monkey, the statistical significance of IDCDs of SMI-32-positive neurons between the ipsilesional and contralesional hemispheres was assessed using a paired *t* test or a Wilcoxon test (according to the data distribution) as the neuronal density was directly compared across the two hemispheres on the same section. In order to compare the interhemispheric morphologic data and the results of the Sholl analysis, a two-way repeated-measures ANOVA with Bonferroni’s *post hoc* test correction wsd used (*p = 0.05, **p = 0.01, ***p = 0.001, ****p = 0.0001). Data are represented as the mean ± SEM, and the significance was set at 95% of confidence.

## Results

Based on a previously available case (Rouiller et al., 1996; intact monkey 93–81), subjected to a unilateral injection of the anterograde tracer BDA in the SMA (F3, focused to the hand area), the bilateral distribution of CS axons was established at cervical level. The majority of CS projection originating from SMA crossed the midline (79.7%), whereas only 20.3% of the BDA positive CS axons were located on the ipsilateral side of the cervical cord (11.7% in the dorsolateral part and 8.6% in the ventromedial part; Fig. 1*A*,*B*). These results suggest that a unilateral cervical cord injury (SCI; Fig. 1*C*) retrogradely impacts more on the contralesional SMA than on the ipsilesional one (connectional diaschisis).

As previously reported (Freund et al., 2006, 2007, 2009; Beaud et al., 2008, 2012; Hoogewoud et al., 2013), eight macaques monkeys (Table 1) were unilaterally injured at C7 level (SCI animals: Mk-CG, Mk-CGa, Mk-CH, Mk-CP, and Mk-CS; SCI + Anti-Nogo-A animals: Mk-AG, Mk-AM, and Mk-AP), and the manual dexterity was quantified using the modified Brinkman board task. After unilateral C7 injury, the ipsilesional manual dexterity was severely affected, followed by a progressive although incomplete functional recovery, reaching a plateau of motor performance a few weeks after the SCI (Fig. 1*D*). Thanks to this longitudinal quantification of the manual dexterity, it was possible to calculate the extent of functional recovery (in %) of the ipsilesional hand and the time duration needed to reach the postlesional plateau (duration of functional recovery; Table 1; Fig. 1*D*).

### Neuronal density in the Layer V of SMA after a SCI

In order to quantify the effect of a unilateral SCI (Fig. 1*C*) on SMA Layer V pyramidal neurons, SMI-32-labeled neurons in the bilateral F3 (SMA-proper) and F6 (pre-SMA) were analyzed histologically in eight macaque monkeys subjected to cervical hemisection and compared with intact monkeys (*n* = 4). First, we quantified the density of SMI-32-positive Layer V neurons in the delimited F3 and F6 areas in both hemispheres along the rostro-caudal axis in coronal sections (Fig. 2*A–D*). In F6 and F3 Layer V, the SMI-32-positive neurons’ densities ranged approximately from 50 cells/mm^3^ to 250 cells/mm^3^ across sections/hemispheres/monkeys in the intact group, whereas in the lesioned monkeys, SMI-32-positive neurons’ densities in SMA Layer V ranged approximately from 100 to 700 cells/mm^3^ (Fig. 2*E*). With respect to those ranges of cellular densities, in the SCI group, there is no obvious difference between the anti-Nogo-A antibody-treated monkeys (*n* = 3) and the control antibody treated animals (*n* = 5). In addition, the range of SMI-32-positive neurons’ densities in the SMA of SCI animals seemed to reach higher values than the intact group (in fact higher than three intact monkeys with low cellular densities in F3, whereas the fourth intact monkey exhibits a cellular density comparable to five monkeys of the SCI group). However, the very large interindividual variability of the histologic staining quality precludes interindividual comparison between the two groups of monkeys and strongly affects the absolute quantification of neuronal density. The quality of SMI-32 staining in three of the four intact monkeys may have been different from in the SCI monkeys, leading to the differences in the absolute numbers of SMI-32-positive neurons between the two groups. On the other hand, the quality of staining does not interfere when the comparison is intraindividual and furthermore restricted to a single histologic section. For this reason, we performed a direct comparison between the hemispheres (left hemisphere compared with the corresponding right hemisphere on the same section).

### IDCD

As expected, intact monkeys presented no significant difference in their IDCDs for the SMI-32-positive neurons in Layer V of F3 (Fig. 3*A*,*B*). On the other hand, the IDCDs of SMI-32-positive neurons were significantly different in six out of eight monkeys subjected to SCI (Fig. 3*B*). However, the IDCD was not systematically biased toward the same hemisphere (ipsilesional vs contralesional; Fig. 3*B*). Four lesioned monkeys (Mk-CG, Mk-CGa, Mk-CP, and Mk-AP) exhibited a significantly higher contralesional density of SMI-32-positive neurons in F3 Layer V, corresponding to a positive IDCD, while two SCI monkeys (Mk-AG and Mk-AM) had a significantly higher ipsilesional density of SMI-32-positive neurons in F3 Layer V, corresponding to a negative IDCD (Fig. 3*A*,*B*).

In order to have an internal control, the cellular density of SMI-32-positive neurons was also quantified in the Layer V of F6 (pre-SMA), a motor cortical area lacking CS neurons (Luppino et al., 1994). The assessment of cellular density in F6 Layer V showed no significant IDCD, both in intact animals and in SCI animals (Fig. 3*C*,*D*). This observation indicates that SMI-32-positive neurons in F6 Layer V were not affected by the SCI, as expected.

Finally, we did not observe significant difference between the calculated IDCD between intact and injured animals neither in F3 nor in F6 (Fig. 3*B’*,*D’*).

### Arborization of Layer V SMI-32-positive neurons in F3 after SCI

**Figure 4.**
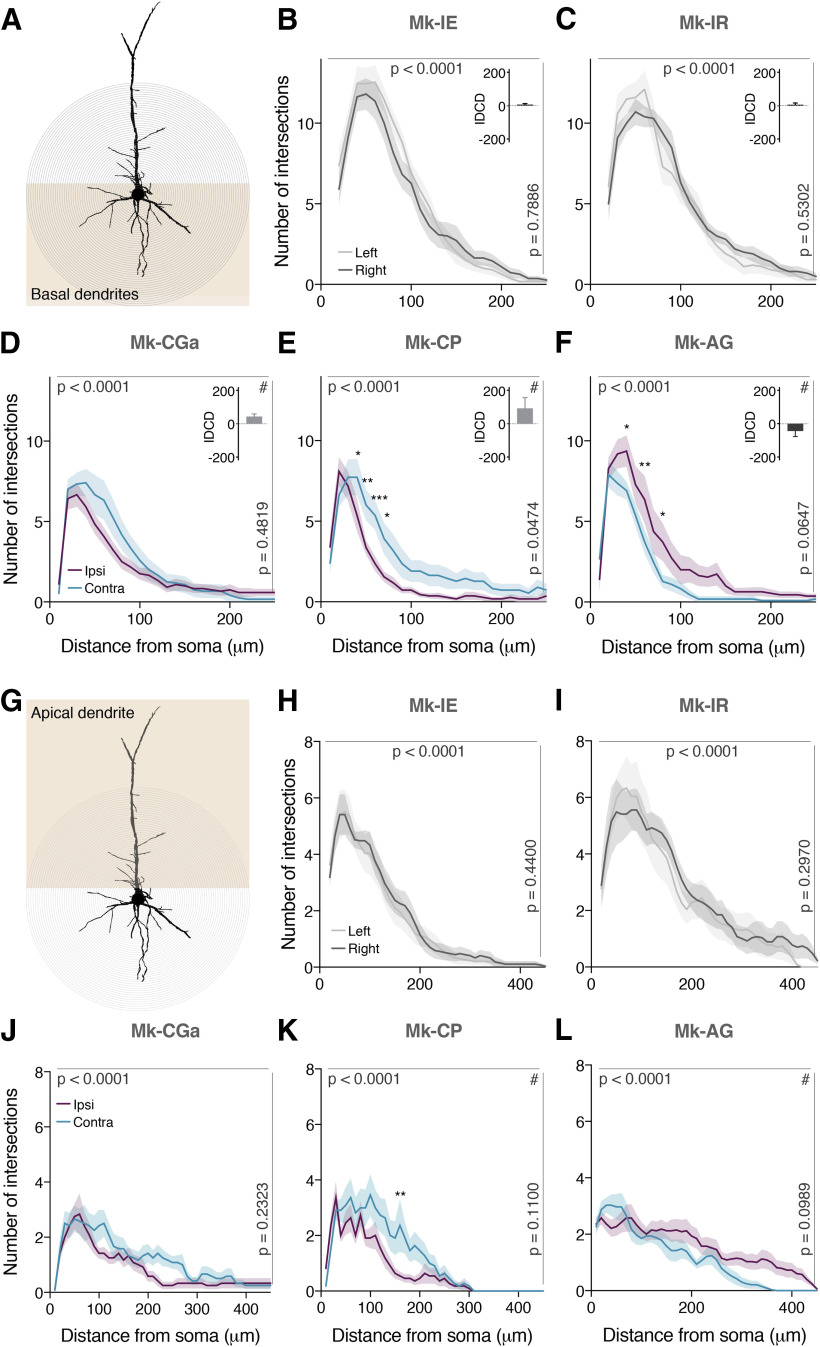
Arborization of Layer V SMI-32-positive neurons in F3 after a SCI. ***A***, Neuronal reconstruction example of basal dendrites of a Layer V SMI-32-positive neuron. ***B–F***, Sholl profiles of basal dendrites of Layer V SMI-32-positive neurons in each hemisphere in two intact monkeys (***A***, ***B***) and in three SCI monkeys (***D–F***). For each monkey, the IDCD are reported in the upper right angle. ***G***, Neuronal reconstruction example of apical dendrite of a Layer V SMI-32-positive neuron. ***H*–*L***, Sholl profiles of apical dendrites of Layer V SMI-32-positive neurons in each hemisphere in two intact monkeys (***H***, ***I***) and in three SCI monkeys (***J–L***). The curves represent the mean intersection values ± SD. As statistical test, a two-way ANOVA wit Bonferroni’s multiple comparison *post hoc* test was performed (*p ≤ 0.05, **p ≤ 0.01, ***p ≤ 0.001). The *p* values for the distance to soma main effect (up) and hemisphere main effect (right) are report for each monkey. # in the upper right angle means a significative *p* value for the interaction between the distance to soma and the hemisphere main effect. *Figure Contributions*: A. Contestabile and M. Lucchini performed the microscopic analysis of the histologic sections. A. Contestabile analyzed the data.

Microstructural changes of the basal (Fig. 4*A*) and apical dendritic (Fig. 4*G*) arborization of SMI-32-positive neurons located in Layer V of F3 was assessed based on a Sholl analysis conducted in two control animals (Mk-IR and Mk-IE) and in three representative lesioned monkeys (Mk-CGa, Mk-CP, and Mk-AG). For all five animals, the Sholl analysis yielded a classical inverted U-shape curve with a tail on the right, corresponding to an increase of dendritic intersection numbers going away from the soma up to a peak, followed by a comparable progressive decrease ending with slower fading at larger distances from the soma. In most analyzed monkeys, the number of dendritic intersections peaked at a distance of ∼50 μm from the soma, both for basal and apical dendrites (Fig. 4). As excepted, no interhemispheric difference was observed in the two control monkeys for both the basal (Fig. 4*B*,*C*) and apical dendrites (Fig. 4*H*,*I*). After SCI, two out of three monkeys exhibited in F3 interhemispheric differences in the basal dendritic arborization (Fig. 4*D–F*). In fact, for Mk-CP and Mk-AG, we observed the same interhemispheric bias consistent with the IDCD bias already observed for Layer V in F3 (Figs. 3*B*, 4*E*,*F*) for the basal dendrites. A comparable tendency (not statistically significant) was observed for Mk-CGa, the third representative lesioned animal. This significant interhemispheric difference of basal dendritic arborizations was verified within a limited distance range from the soma, going from 40 to 80 μm (Fig. 4*E*,*F*). Interestingly, and in contrast to the basal dendrites, the apical dendrites exhibited clearly less pronounced interhemispheric difference in their numbers of dendritic intersections (Fig. 4*J–L*).

### Relationship of IDCDs in F3 with percentage of hemisection extent, duration and extent of functional recovery after SCI

**Figure 5.**
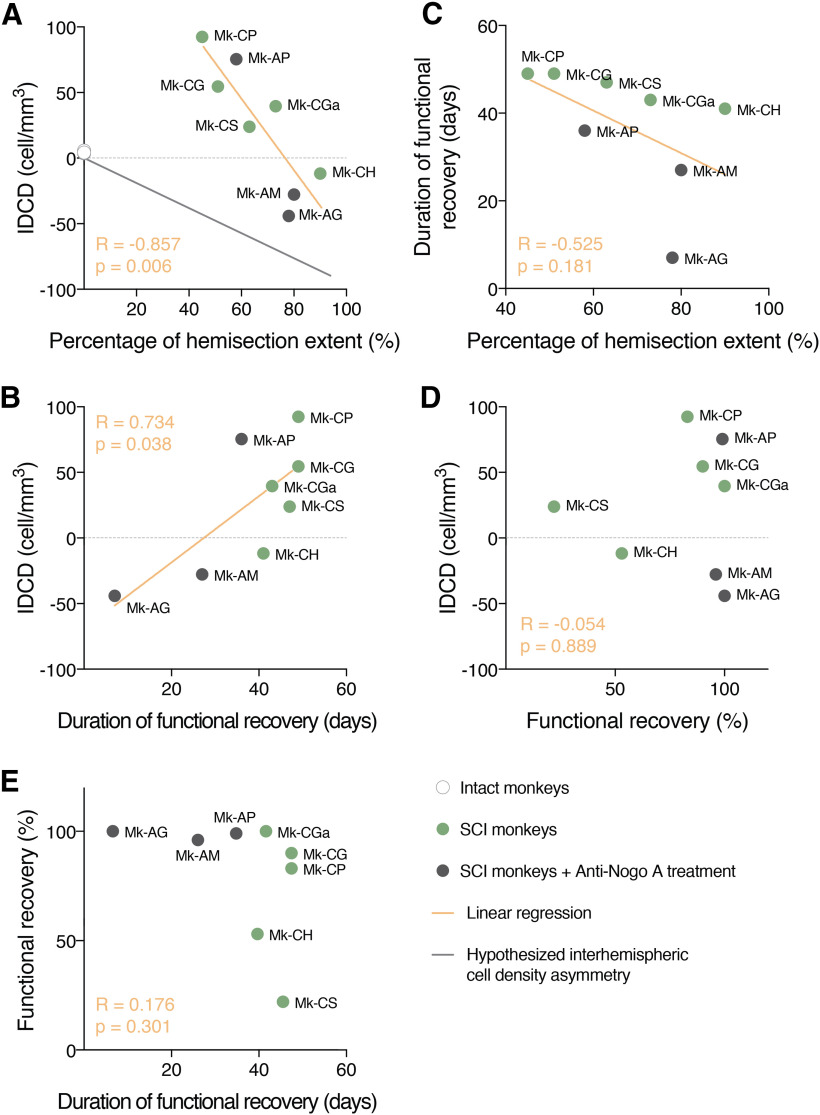
Relationship of IDCD, proportion of hemisection extent, duration of recovery and percentage of functional recovery. ***A***, The IDCD in F3 was plotted in function of the proportion of hemisection extent. The gray line represents the hypothesized interhemispheric cell density asymmetry (see Introduction). ***B***, The IDCD in F3 was plotted in function of the duration of functional recovery. ***C***, The duration of functional recovery was plotted in function of the proportion of hemisection extent. ***D***, The IDCD in F3 was plotted in function of the functional recovery. ***E***, The functional recovery of the animals was plotted in function of the duration of functional recovery. Intact (in white), SCI (light gray), and SCI-treated (dark gray) animals are divided by color code. In each graph, the *R* and the *p* value of the linear regression are reported. *Figure Contributions*: A. Contestabile, R. Colangiulo, E. M. Rouiller, and E. Schmidlin performed the experiments and collected the data. A. Contestabile analyzed the data.

Are the morphologic interhemispheric changes on SMI-32-positive neurons in F3 Layer V induced by SCI (Figs. 2, 3) related to the characteristics of the lesion (size) and/or the properties of functional recovery? To this aim, IDCDs of SMI-32 neurons in F3 were plotted as a function of lesion extent (percentage of hemisection), durations of functional recovery and extent in percent of functional recovery (Fig. 5*A–C*). Interestingly, IDCDs in F3 Layer V were significantly inversely correlated with the size of the SCI, expressed as the percentage of hemisection (*R* = –0.857, *p* = 0.006; Fig. 5*A*), independently of the presence/absence of anti-Nogo-A antibody treatment. A similar inverse correlation was found between IDCDs and lesion size in F3 after unilateral M1 lesion (Contestabile et al., 2018). In both cases, these correlation data indicate that a small lesion (ipsilateral in the case of cortical lesion or contralateral in case of SCI) is associated with a largely positive IDCD, whereas large lesions are associated to negative IDCDs.

The relationship between the median IDCD values and the duration of functional recovery from SCI (Fig. 5*B*) was then investigated, based on behavioral data derived from the modified Brinkman board task: the IDCDs in F3 Layer V were correlated with the duration of functional recovery (*R* = 0.734, *p* = 0.038; Fig. 5*B*), again independently of the presence/absence of treatment. Interestingly, there was no statistically significant correlation between the duration of functional recovery from SCI and the hemisection extent of the spinal cord (*R* = –0.525, *p* = 0.181; Fig. 5*C*). However, the three SCI monkeys which received the anti-Nogo-A antibody treatment recovered faster (shorter time to reach the postlesional plateau) than the control antibody-treated monkeys, a difference already visible in Figure 5*B*. In contrast to the parameters lesion extent and duration of functional recovery, IDCDs in F3 Layer V were not correlated with the parameter extent of functional recovery (expressed in %; *R* = 0.054, *p* = 0.889; Fig. 5*D*), assessed with the total score of pellets retrieval in the modified Brinkman board task. Finally, as observed in Contestabile et al. (2018); no correlation was observed between the percentage of functional recovery in the modified Brinkman board task and duration of functional recovery (*R* = 0.176, *p* = 0.301; Fig. 5*E*), suggesting that a longer duration of functional recovery did not mean a better functional recovery.

## Discussion

The major conclusion of the present study is that a unilateral cervical cord hemisection retrogradely induced an interhemispheric asymmetry of SMI-32 staining in F3 Layer V in non-human primates, which is correlated with the extent of the SCI as well as the duration of functional recovery, but not with the extent (percentage) of the functional recovery. These conclusions are coherent with the observations described recently by our laboratory showing similar results in F3 Layer V in SMA after a unilateral M1 lesion (Contestabile et al., 2018). A coherent correlation between IDCDs and lesion size as well as duration of functional recovery (but not extent of recovery) were observed in this study. In other words, the interhemispheric effects on SMI-32 staining in F3 Layer V observed after M1 lesion (Contestabile et al., 2018) can be generalized to another type of unilateral lesion, namely a cervical cord hemisection. If the morphologic interhemispheric changes reflect variable balanced contributions of the two hemispheres in the process of functional recovery, then comparable mechanisms of functional recovery at cortical level may take place, regardless of the site of lesion (cortical or spinal), at least for injury restricted to one side of the brain or of the spinal cord.

The lateralization of the unilateral lesion, either in M1 or at cervical cord level, is of importance. The computation formula to establish the IDCD was chosen to be reversed in the present study (cervical cord lesion) as compared with our recent report on unilateral M1 lesion (Contestabile et al., 2018). The reason for such reversal is that the unilateral M1 lesion was on the same hemisphere as the F3 area, which exhibits (before the lesion) the quantitatively predominant corticocortical connection with the injured M1, whereas the transcortical connection from the opposite F3 is less strong (Contestabile et al., 2018, their Fig. 1). In case of cervical cord hemisection, the predominant CS projection from F3 is crossed (originating mainly from the opposite hemisphere), reason why the IDCD formula was reversed (see Materials and Methods). Because of this reversal, then the correlation plots in the M1 lesion model (Contestabile et al., 2018; their Fig. 5) and in the present study (Fig. 5) should appear similar, if the same correlations are verified. This is indeed the case for the correlation between IDCDs and the lesion extent (compare Contestabile et al., 2018, their Fig. 5*B* with the present Fig. 5*A*). In contrast, although there is a correlation in both lesion models between IDCDs and duration of functional recovery, the correlation was inverse in the M1 lesion model (Contestabile et al., 2018; their Fig. 5*F*) and positive in the present SCI model (Fig. 5*B*). As argued above, because of the reversal of the IDCD formula, we would have intuitively expected a similar correlation direction. This is not the case, suggesting that the mechanisms of recovery reflected by the correlation between IDCD and duration of functional recovery may be independent of the lateralization of the unilateral lesion, either cortical or cervical.

### Connectional diaschisis

The results obtained in Contestabile et al. (2018), and in this study could be linked with the old concept of diaschisis (von Monakow, 1914), defined as a “loss of function and electrical activity in an area of the brain because of a lesion in a remote area that is neuronally connected with it,” although it corresponds more to a “connectional diaschisis” (Carrera and Tononi, 2014; see Introduction). The unilateral SCI may have affected the morphology, phenotype and cellular expression of Layer V neurons in the contralesional hemisphere in a different manner with respect to the neurons localized in the ipsilesional hemisphere. This interhemispheric difference in phenotype could have influenced the affinity for the SMI-32 antibody and explain the observed interhemispheric asymmetry of density of SMI-32-positive Layer V neurons. This interpretation is moreover supported by the neuronal reconstruction. In fact, the directions of interhemispheric differences in dendritic arborization complexity (Fig. 4) were consistent with the direction of the IDCD, especially in the arborization of the basal dendrites, which are more implicated in the integration of the neuronal response and have a strong effect on action potential output because of their direct attachment to the cell body and the proximity to the axon (Nevian et al., 2007; Zhou et al., 2008). The changes of dendritic arborization following SCI can be linked to the recently published observation depicting that axotomy of peripheral motor projections induce changes in the dendritic arborization of M1 pyramidal neurons in the rodent model submitted to a permanent lesion of the facial nerve (Urrego et al., 2015). In another recent study aiming at measuring the structural changes in the CNS of human patients suffering from degenerative pathology affecting interaction between cortical motoneurons and spinal motoneurons such as amyotrophic lateral sclerosis (ALS) or dementia, the authors observed a degeneration of the apical dendrites of pyramidal neurons located in Layer V of M1 (Betz’s cells as described in the paper), to a larger extent in patients suffering from ALS (Genç et al., 2017). These results are consistent with a lower dendritic arborization observed in the contralesional SMA (Fig. 4*G*,*L*) in animals having a larger extent of SCI, as ALS is a major degenerative disease affecting a majority of motoneurons in the ventral horn of the spinal cord.

Interestingly, it seems that anti-Nogo-A antibody treatment does not have an effect on the observed interhemispheric difference in phenotype. The three treated animals showed results that are comparable to only lesioned animals. Neutralization of Nogo-A was found to promote sprouting of CS axons in macaque monkeys and to lead to a complete recovery of the manual dexterity after a unilateral hemisection lesion irrespectively on the lesion extent (Freund et al., 2006, 2007, 2009). However, it is possible that anti-Nogo-A antibody treatment could not preserve the phenotype of F3 Layer V pyramidal neurons since the SCI immediately affect the neuronal connection and altered the phenotype of the CS neurons in an irreversible manner.

### A possible role of the ipsilesional hemisphere during the functional recovery from SCI

The correlation between IDCD asymmetry and the duration of functional recovery is also reminiscent of the controversy related to the respective contributions of the ipsilesional versus contralesional hemispheres in the functional recovery following a unilateral SCI. Previous studies in rodents (Ghosh et al., 2009) and humans (Lundell et al., 2011) demonstrated that the ipsilesional hemisphere underwent a profound reorganization of the motor areas after cervical SCI. Moreover, these previous findings suggest a correlation between brain activity, duration and extent of functional recovery and are consistent with our results. In fact, they revealed that monkeys with larger lesion tend to show shorter duration of functional recovery and negative IDCD suggesting a more prominent reorganization and a predominant contribution of the ipsilesional hemisphere (as compared with the contralesional one), immediately after severe SCI. On the other hand, the data also show that a more moderate lesion leads to a higher SMI-32-positive neurons density in the contralesional hemisphere suggesting a more important reorganization of the hemisphere predominantly affected by the spinal hemisection. Interestingly, previous studies reported an upregulation of the expression of Sema3A and NRP-1 in motoneurons located in the contralesional hemisphere after SCI in rats (De Winter et al., 2002; Hashimoto et al., 2004). It is likely that neurons whose descending fibers were transected upregulated the production of proteins involved in the regulation of axonal re-growth and maintenance. This phenomenon may possibly be correlated with the higher density of neurons in the contralesional hemisphere that express SMI-32, a major component of the neuronal cytoskeleton providing structural support to the axon. However, in a recent study based on meta-analysis of fMRI data assessing the possible reorganization of different regions of the cerebral cortex after SCI in human patients, the authors showed changes in bilateral SMA, but were unable to specify the type of change or potential role in the functional recovery (Wang et al., 2019).

### Limitations

The present study involves a limited number of monkeys subjected to SCI (*n* = 8), five of them received a control antibody, while three monkeys were subjected to anti-Nogo-A antibody treatment, as one may reasonably expect from a non-human primate study, mostly for ethical reasons. However, despite the limited number of monkeys, the data are internally coherent (e.g., F3 vs F6) and statistically significant correlations of IDCDs with lesion extent and duration of functional recovery emerged (Fig. 5). Furthermore, these correlation data are fully consistent with previous data derived from another pool of monkeys (*n* = 9) subjected to another type of unilateral lesion (M1) affecting the CS projection (Contestabile et al., 2018).

An important limitation of this study is because of the impossibility to compare the absolute values of SMI-32-positive neurons’ densities across animals. Although the raw data seems to show a higher density of SMI-32 neurons in both hemispheres of SCI monkeys as compared with intact animals, such interindividual comparison between the two groups of monkeys is problematic because of a very large interindividual variability of the histologic staining quality, strongly affecting the absolute quantification of neuronal density. The quality of SMI-32 staining in three of the four intact monkeys may have been different (lower) than in the SCI monkeys, leading to the differences in the absolute numbers of SMI-32-positive neurons between the two groups. In one of the intact monkeys (Mk-IC), the absolute cellular density is comparable to that of five of the SCI monkeys, corresponding thus to a partial overlap between the two groups (which are not statistically different with respect to their IDCDs in F3 and F6 (Fig. 3*B*,*D*). On the other hand, the quality of staining does not interfere when the comparison is intraindividual and furthermore restricted to a single histologic section (left hemisphere compared with the corresponding right hemisphere on the same section, both hemispheres processed histologically in the very same way and at the same time point).

Further limitations were related to the impossibility in some monkeys to perform the cell counting and consecutive IDCD analysis in F6 because of the lack of brain tissue. Finally, the histologic condition of some SMI-32 sections was not optimal to perform the neuronal dendritic reconstruction and for this reason the morphologic Sholl analysis was limited to five animals (Fig. 4).

## Conclusion

Altogether, the current study on SCI and the recent investigation on M1 lesion (Contestabile et al., 2018) both show that a unilateral lesion affecting the CS projection system, either at its origin (in M1) or along its trajectory (at cervical level) results in a variable and adaptable interhemispheric balance of F3 Layer V pyramidal neurons, detected with the marker SMI-32, in a manner which is systematically correlated with the extent of the lesion as well as the duration of the functional recovery of manual dexterity. These observations suggest that the controversy on which hemisphere (ipsilesional vs contralesional) is involved in the functional recovery may be resolved by considering a contribution of both, however in an adaptable and finely tuned balance depending on the lesion properties (e.g., extent) and evolving with time during the recovery, the duration of the latter being a crucial factor in this process.

## Synthesis

Reviewing Editor: Karl Herrup, University of Pittsburgh School of Medicine Decisions are customarily a result of the Reviewing Editor and the peer reviewers coming together and discussing their recommendations until a consensus is reached. When revisions are invited, a fact-based synthesis statement explaining their decision and outlining what is needed to prepare a revision will be listed below. The following reviewer(s) agreed to reveal their identity: NONE. Note: If this manuscript was transferred from JNeurosci and a decision was made to accept the manuscript without peer review, a brief statement to this effect will instead be what is listed below.

The work is modest in scale due to the small number of subjects, but the question is important and the data that emerge offer some compelling insights. There is room for improvement before final acceptance, however.

Two clarifications are needed: (1) Were the monkeys actively trained for the task, or they were just tested at the time of daily test? and (2) What does the increase in the density volume of SMI-32 positive cell mean? Does that mean some metabolic change in the layer V pyramidal cells? Increase/decrease in the neurites and volume increase/decrease in the glial cells expanded the region, which in turn decreased/increased the density? Without argument on these, the readers cannot interpret what really happened in the SMA of these monkeys. The glial cell question is a a particularly important one since no other cell type except for neuron was examined.

The authors should also fix two other minor problems.

Line 305; what does Fig.2E-P mean? There is no labeling like “P” in the figure.

Line 337-338; what does the “animal groups” here indicate?

No additional work is required, but if it is possible to obtain data on the response of the non-neuronal cells, this would greatly strengthen the work.
